# Glycine Protects H9C2 Cardiomyocytes from High Glucose- and Hypoxia/Reoxygenation-Induced Injury via Inhibiting PKC*β*2 Activation and Improving Mitochondrial Quality

**DOI:** 10.1155/2018/9502895

**Published:** 2018-04-04

**Authors:** Yuan Zhang, Wating Su, Qiongxia Zhang, Jinjin Xu, Huimin Liu, Jun Luo, Liying Zhan, Zhongyuan Xia, Shaoqing Lei

**Affiliations:** Department of Anaesthesiology, Renmin Hospital of Wuhan University, Wuhan, China

## Abstract

**Background:**

Patients with diabetes are more vulnerable to myocardial ischemia reperfusion injury (IRI), which is involved in PKC*β*2 activation and mitochondrial dysfunction. Glycine has been documented as a cytoprotective agent to attenuate diabetes-related abnormalities and reduce myocardial IRI, but the underlying mechanisms are still unclear. We determined whether glycine could attenuate high glucose- (HG-) and hypoxia/reoxygenation- (H/R-) induced injury by inhibiting PKC*β*2 activation and improving mitochondrial quality in cultured H9C2 cells.

**Methods:**

H9C2 cells were either exposed to low glucose (LG) or HG conditions with or without treatment of glycine or CGP53353 (a selective inhibitor of PKC*β*2) for 48 h, then subjected to 4 h of hypoxia followed by 2 h of reoxygenation (H/R). Cell viability, lactate dehydrogenase (LDH) release, mitochondrial membrane potential (MMP), superoxide dismutase (SOD) activity, and malondialdehyde (MDA) concentration were detected using corresponding commercial kits. Mitochondrial quality control-related proteins (LC-3II, Mfn-2, and Cyt-C) and PKC*β*2 activation were detected by Western blot.

**Results:**

HG stimulation significantly decreased cell viability and SOD activity and increased LDH release, MDA production, and PKC*β*2 activation as compared to LG group, all of which changes were further increased by H/R insult. Glycine or CGP53353 treatment significantly reduced the increase of LDH release, MDA production, PKC*β*2 activation, and Cyt-C expression and the decrease of cell viability, SOD activity, MMP, Mfn-2 expression, and LC-3II/LC-3I ratio induced by HG and H/R stimulation.

**Conclusions:**

Supplementary glycine protects H9C2 cells from HG- and H/R-induced cellular injury by suppressing PKC*β*2 activation and improving mitochondria quality.

## 1. Introduction

Acute myocardial infarction (AMI) is one of the leading causes of death in patients with diabetes [1]. Hyperglycemia-induced oxidative stress plays a vital role in the development and progression of AMI [2]. Reperfusion therapies (e.g., thrombolysis, angioplasty, stent placement, and coronary artery bypass grafting) when applied expeditiously may limit cardiac dysfunction and infarct size, but reperfusion also elicits more tissue injury, termed as ischemia-reperfusion injury (IRI). Increasing evidence has suggested that diabetic hearts are more vulnerable to IRI [3]. In diabetic condition, excessive reactive oxidative species (ROS) production induced by hyperglycemia may decrease mitochondrial membrane potential (MMP) and increase mitochondrial permeability transition pore (MPTP) opening resulting in mitochondrial dysfunction, which may in turn increase ROS production and exacerbate myocardial IRI [4]. Additionally, a burst production of ROS during reperfusion has been demonstrated to be an important factor exacerbating myocardial IRI [5]. Thus, effective means attenuating ROS formation may confer cardioprotection against myocardial IRI.

It is well established that chronic hyperglycemia-induced abnormal activation of PKC, a family of enzymes controlling the functions of other proteins, is associated with the development of diabetic cardiovascular complications [6, 7]. Among the various isoforms of PKC, PKC*β* isoforms are the most frequently implicated in diabetic cardiovascular complications [7]. It has been demonstrated that PKC*β* activation may contribute to diabetic abnormalities by increasing ROS production derived from NADPH oxidase activation [8], negatively modulates mitochondrial energy status, and inhibits autophagy [9]. In our previous studies, PKC*β*2 but not PKC*β*1 was found to be excessively activated in diabetic hearts, and selective inhibition of PKC*β*2 activation might attenuate ROS production and ameliorate myocardial IRI [10, 11]. In addition, PKC*β*2 activation has been identified to influence the activity of the tricarboxylic acid cycle and contribute to impaired mitochondrial function in hearts from type 1 diabetic rats [12]. Emerging reports indicated transient brain IRI-induced translocation of PKC*β*2 to mitochondria and consequent overactivation ameliorated mitophagy (a critical mechanism of mitochondria quality control) dysfunction and neuronal death in hippocampus ischemia-resistant regions of gerbils [13]. All these findings suggest that PKC*β*2 may play an important role in mitochondrial quality control. However, little is known about the relationship between impaired mitochondria and PKC*β*2 activation in cardiomyocytes under hyperglycemic condition, especially with acute ischemia-reperfusion stimulation.

Glycine, a nonessential amino acid, plays an important role in metabolic regulation, antioxidative reactions, and neurological function [14]. It has been documented as a cytoprotective agent to attenuate diabetes-related abnormalities and reduce myocardial IRI [14, 15]. However, whether glycine exerts a beneficial effect on ischemic cell injury under hypoglycemic condition is unknown. In the present study, we hypothesized that glycine protects H9C2 cardiomyocytes from high glucose- (HG-) and hypoxia/reoxygenation- (H/R-) induced injury by inhibiting PKC*β*2 activation and improving mitochondrial quality.

## 2. Materials and Methods

### 2.1. Cell Culture

H9C2 cells (rat embryonic ventricular myocytes) were obtained from the Cell Bank of the Chinese Academy of Sciences (Shanghai, China) and were routinely cultured in complete DMEM media containing 5 mM glucose and L-glutamine (Gibco Laboratories, USA) and supplemented with 10% fetal bovine serum (Gibco Laboratories, USA) and 1% antibiotics (100 U/mL penicillin and 100 mg/mL streptomycin (Gibco Laboratories, USA)). The cell lines were cultured in T75 flasks (Sigma) at 37°C in a humidified atmosphere, with 5% CO_2_. Media were replaced every 2 to 3 days and cells were split when they approximately reach 80% confluence. H9C2 in logarithmic growth phase were trypsinized and seeded in 6- or 96-well plates (Sigma) for follow-up experiments.

### 2.2. HG and H/R Procedure

When cells have grown to 50% confluence in 6-well plates, 50% glucose injection was used at a final concentration of 30 mM glucose for the HG procedure. After exposure to HG for 48 h, the H/R procedure was performed. For hypoxia exposure, cells were maintained under anoxic conditions in chambers gassed with a mixture of 95% N_2_, 5% CO_2_, and 1% O_2_ at 37°C for 4 h. For reoxygenation, plates were removed from the anoxic chamber to a normoxic chamber for 2 h.

### 2.3. Drug Treatment

Glycine (Sigma) was made up in deionized water and used at a final concentration of 140 *μ*M [16]. CGP53353 (Sigma), a specific inhibitor of PKC*β*2, was dissolved in dimethyl sulfoxide (DMSO) and used at a concentration of 1 *μ*M [10].

### 2.4. Determination of Cytotoxicity

Cytotoxicity was assessed by measurement of cell viability and LDH release in the medium using CCK-8 and LDH assay kits, respectively (Nanjing Jiancheng Bioengineering Institute, China). For CCK-8 assay, cells in 96-well plates were treated according to the experimental procedure and then incubated with 10% CCK-8 reagents and 90% fresh DMEM media for 60 min at 37°C before the assay was performed. Media in 6-well plates were centrifugalized to gather supernatant for LDH release test. All operations were carried out according to the manufacturer's instructions.

### 2.5. Determination of Lipid Peroxidation and Superoxide Dismutase (SOD) Activity

The content of malondialdehyde (MDA), a marker of lipid peroxidation, was determined to assess oxidative injury of H9C2 cells subjected to HG and H/R. We also evaluated the antioxidant content by measuring SOD activity. After homogenizing on ice in normal saline, the levels of MDA and SOD activity in the supernatants were determined using MDA and SOD activity assay kits according to the manufacturer's instructions (Nanjing Jiancheng Bioengineering Institute, China).

### 2.6. Assessment of Mitochondrial Membrane Potential (MMP)

MMP was assessed by JC-1 (Beyotime, China) staining according to the experimental protocol. Briefly, H9C2 cells were cultured to ~80% confluence in a 6-well plate. Then, cells were washed 3 times with precold PBS. After that, cells were incubated with JC-1 working liquid and complete medium for 20 min at 37°C. Cells were subsequently washed twice with JC-1 buffer and the media replaced with fresh media. Finally, cells were scanned with epifluorescence. The monomeric and aggregated forms of JC-1 emit green and red fluorescence, respectively, which correspondingly represented depolarized and normal MMP.

### 2.7. Western Blot Analysis

Cells were lysed using RIPA buffer containing protease inhibitor mixture (Beyotime) and protein concentration determined using the BCA kit (Beyotime). Equal amounts of proteins were loaded in SDS-PAGE (5%-10%-15% acrylamide). After electrophoretic separation, proteins were transferred to polyvinylidene difluoride (PVDF) membrane and subsequently blocked for 1 h at room temperature in 5% no-fat milk, followed by overnight incubation with rabbit anti-Mfn-2 (1 : 1000 dilution, Abcam company, USA) or anti-LC3, anti-phospho-PKC*β*2, and anti-PKC*β*2, (1 : 1000 dilution, Cell Signaling Technology, USA) in 4°C shaker. Membranes were subsequently washed with Tris-buffered saline with 0.1% Tween 20 (TBST) for 30 min and incubated with the appropriate fluorescent secondary antibodies (1 : 10000 dilution, Cell Signaling Technology, USA) for 1 h at room temperature. Then, membranes were washed again with TBST. Finally, the Odyssey Infrared Imaging System (USA, LI-COR) was used to detect fluorescent signals and capture digital images. The images were analyzed using Odyssey Application Software 3.0 to obtain the integrated intensities, followed by linear regression of the intensity data.

### 2.8. Statistical Analysis

All statistical analyses were performed using GraphPad Prism 6.0 (GraphPad Software Inc., San Diego, CA, USA). For statistical analysis, one- or two-way repeated-measures ANOVA followed by Tukey's post hoc test in grouped values was performed wherever applicable. Statistical significance was set at *P* < 0.05 and the data are represented as mean *±* SD.

## 3. Results

### 3.1. HG-Treated H9C2 Cells Are More Vulnerable to H/R Injury

**Figure 1:**
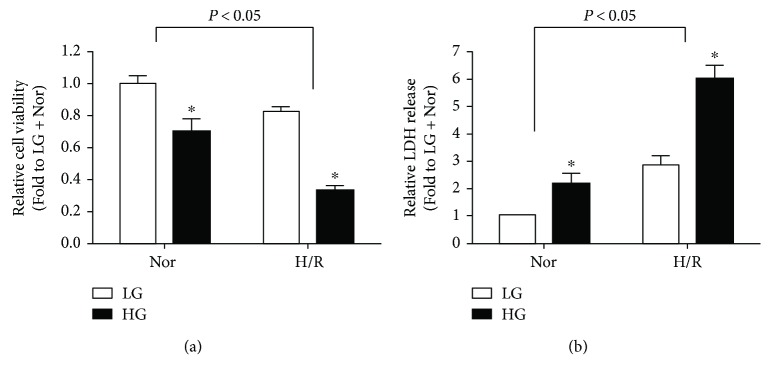
Effects of high glucose (HG) and hypoxia/reoxygenation (H/R) on cellular viability (a) and the release of LDH (b) in H9C2 cells. All the results are expressed as mean ± SD (*n* = 8). Differences were determined by using two-way ANOVA followed by Tukey's post hoc test. ^∗^ *P* < 0.05 versus LG (low glucose) group in normoxic or hypoxic conditions.

In the present study, we first observed cytotoxicity by assaying cell viability and LDH release in cultured H9C2 cells under LG and HG conditions with or without H/R stimulation. As shown in Figures 1(a) and 1(b), H9C2 cells exposed to HG stimulation exhibited decreased cell viability and increased LDH release as compared with LG group in normoxic conditions, and these alterations were further increased by H/R insult as compared with those in the corresponding normoxic groups, indicating that HG-treated H9C2 cells are more vulnerable to H/R injury.

### 3.2. HG- and H/R-Induced H9C2 Cell Injury Is Associated with Excessive Oxidative Stress and PKC*β*2 Activation

**Figure 2:**
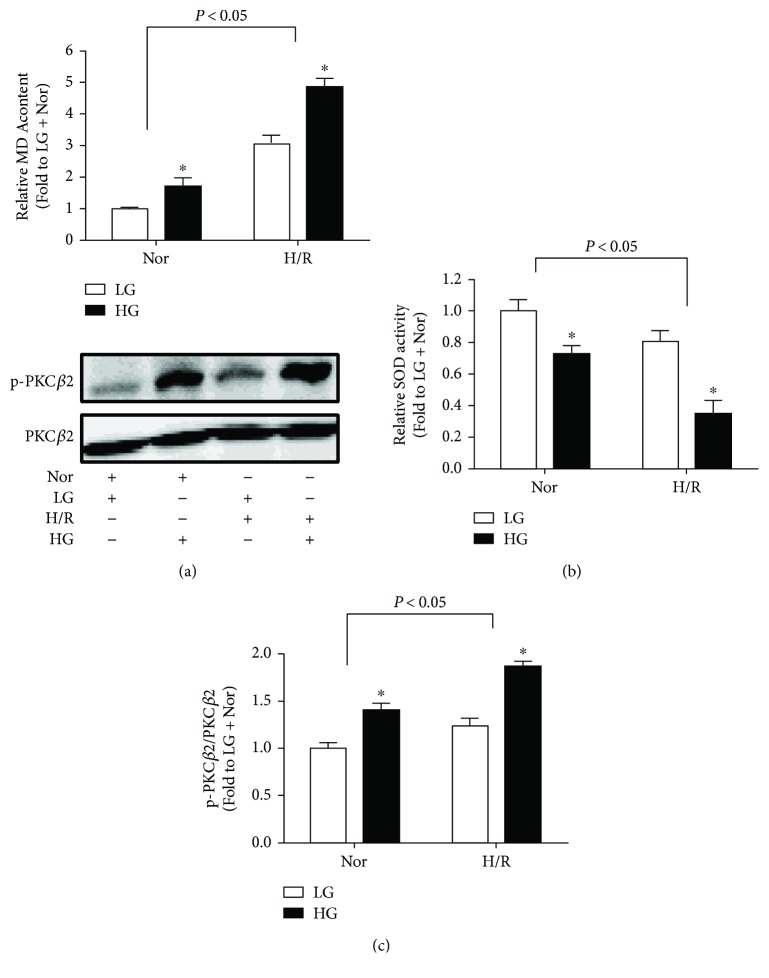
Effects of high glucose (HG) and hypoxia/reoxygenation (H/R) on MDA production (a), SOD activity (b), and PKC*β*2 activation (c) in H9C2 cells. All the results are expressed as mean ± SD (*n* = 8). Differences were determined by using two-way ANOVA followed by Tukey's post hoc test. ^∗^ *P* < 0.05 versus LG (low glucose) group in normoxic or hypoxic conditions.

As the important role of oxidative stress in the development and progression of IRI [3], we then measured the biochemical markers of oxidative stress including MDA production and SOD activity in the cell homogenization. HG stimulation significantly increased MDA production as compared with that in LG group, which was further increased by H/R insult (Figure 2(a)). In comparison, SOD activity was significantly decreased by HG stimulation, and H/R insult further decreased SOD activity (Figure 2(b)).

Hyperglycemia-induced PKC*β*2 activation played an important role in myocardial IRI in diabetes [10, 11]. Thus, we detected the protein expression of PKC*β*2 and its phosphorylation. HG stimulation moderately increased PKC*β*2 phosphorylation at Thr642 (data not shown), but its increase in phosphorylation at Ser660 was most profound without affecting total PKC*β*2 expression, which results in a significantly increased ratio of phosphorylated PKC*β*2 to total PKC*β*2 (Figure 2(c)), indicating PKC*β*2 activation. H/R stimulation further increased the ratio of phosphorylated PKC*β*2 to total PKC*β*2.

### 3.3. Glycine Treatment Could Attenuate HG- and H/R-Induced H9C2 Cell Injury, Which Is Involved in the Inhibition of PKC*β*2 Activation

**Figure 3:**
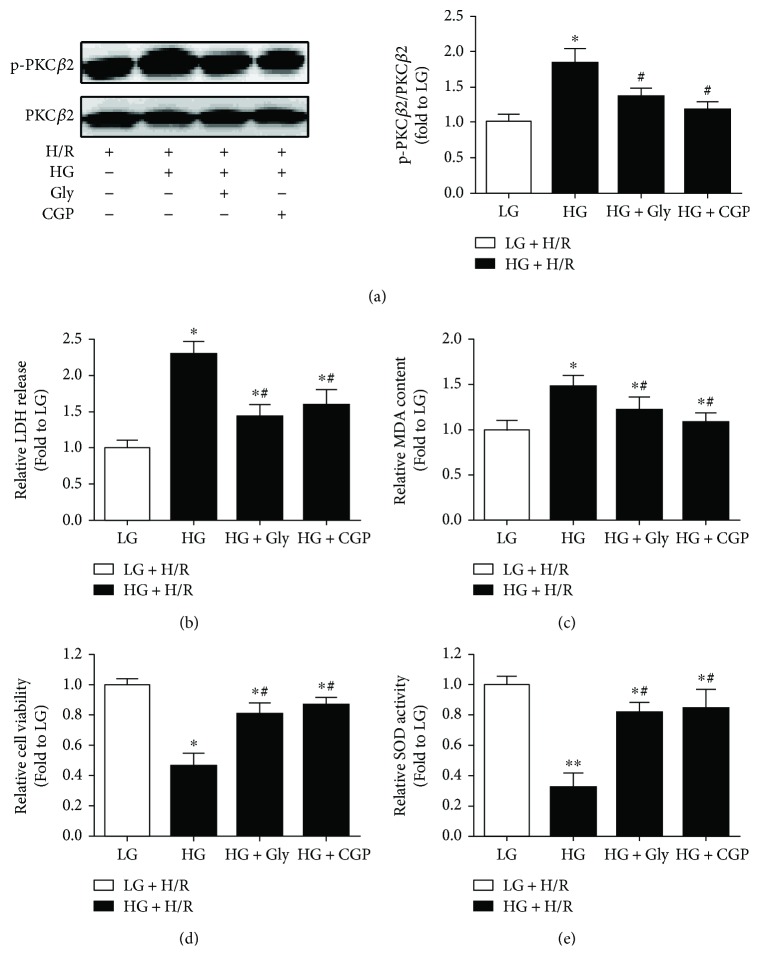
Effects of glycine (Gly) and CGP53353 (CGP) on PKC*β*2 activation (a), LDH release (b), MDA production (c), cellular viability (d), and SOD activity (e) in H9C2 cells subjected to high glucose (HG) and hypoxia/reoxygenation (H/R) insult. All the results are expressed as mean ± SD (*n* = 8). Differences were determined by using one-way ANOVA followed by Tukey's post hoc test. ^∗^ *P* < 0.05, ^∗∗^ *P* < 0.01 versus LG (low glucose) + H/R groups, and ^#^ *P* < 0.05 versus HG + H/R.

We next investigated the treatment effects of glycine on PKC*β*2 activation in H9C2 cells subjected to HG and H/R stimulation. As shown in Figure 3(a), the excessive activation of PKC*β*2 activation induced by HG and H/R stimulation was well inhibited by CGP (a selective inhibitor of PKC*β*2). By contrast, glycine treatment also significantly attenuated PKC*β*2 activation. We also investigated the treatment effects of glycine and CGP on cytotoxicity and oxidative stress. As shown in Figures 3(b) and 3(c), the increased LDH release and MDA production induced by HG and H/R stimulation were significantly decreased by glycine treatment; similar effects were shown in the treatment of CGP. In addition, the decreased cell viability and SOD activity induced by HG and H/R were significantly increased by glycine or CGP treatment (Figures 3(d) and 3(e)).

### 3.4. Effects of Glycine on Mitochondrial Membrane Potential (MMP)

**Figure 4:**
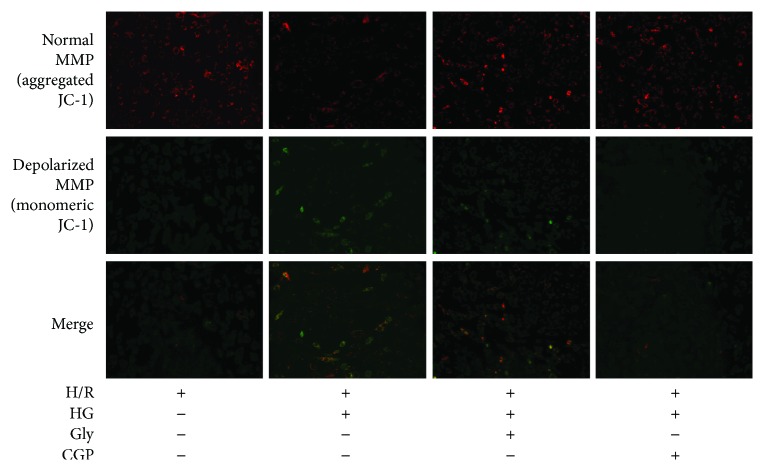
Effects of glycine (Gly) and CGP53353 (CGP) on mitochondrial membrane potential (MMP) in H9C2 cells subjected to high glucose (HG) and hypoxia/reoxygenation (H/R) insult.

Mitochondria are the main site of oxidative stress, and MMP depolarization has been implicated in mitophagy due to mitochondrial dysfunction [17]. Considering the possible source of ROS, MMP was evaluated by JC-1 staining. As shown in Figure 4, the vast H9C2 cells under LG and normoxic conditions emitted bright red fluorescence, whereas a diffuse green fluorescence in majority of HG- or H/R-treated H9C2 cells was observed indicating mitochondrial damage. We observed further decline in MMP when cells were treated with HG followed by H/R. By contrast, the cells preadministered with CGP or glycine exhibited a significant preservation of red fluorescence.

### 3.5. Effects of Glycine on the Protein Expression of LC-3II, Cyt-C, and Mfn-2

**Figure 5:**
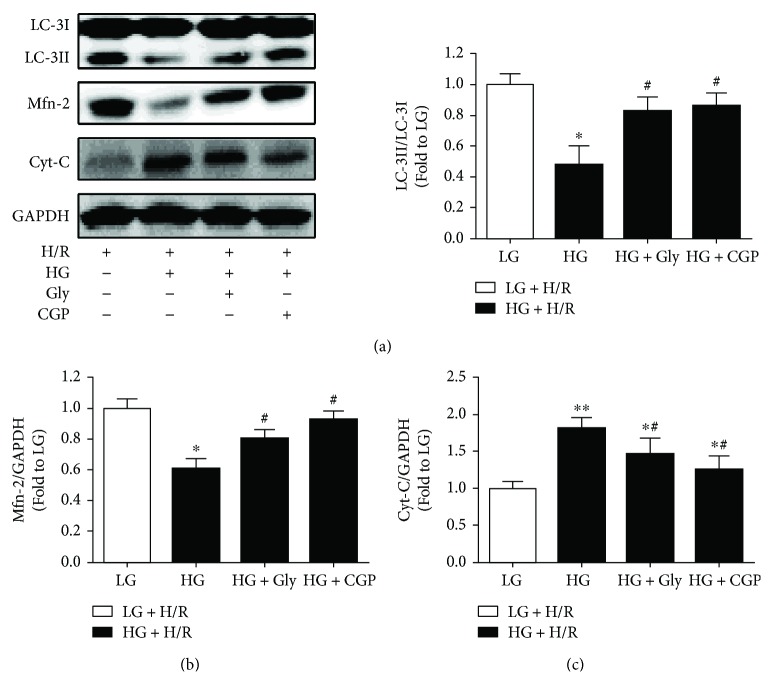
Effects of glycine (Gly) and CGP53353 (CGP) on LC-3II (a), Mfn-2 (b), and Cyt-C (c) expression in H9C2 cells subjected to high glucose (HG) and hypoxia/reoxygenation (H/R) insult. All the results are expressed as mean ± SD (*n* = 8). Differences were determined by using one-way ANOVA followed by Tukey's post hoc test. ^∗^ *P* < 0.05, ^∗∗^ *P* < 0.01 versus LG (low glucose) + HR groups, and ^#^ *P* < 0.05 versus HG + H/R.

We then evaluated the autophagy status by detecting the ratio of LC-3II/LC-3I, and mitochondria quality-control proteins including Cyt-C and Mfn-2 expression were also detected in H9C2 cells subjected to HG and H/R stimulation. As shown in Figures 5(a) and 5(b), both LC-3II/LC-3I ratio and Mfn-2 expression in H9C2 cells under LG and H/R condition were much lower than that in HG and H/R group, which were increased by treatment with glycine or CGP. By contrast, HG and H/R significantly increased Cyt-C expression as compared to that in LG and H/R group, and this increased Cyt-C expression was significantly reduced by either glycine or CGP treatment (Figure 5(c)).

## 4. Discussion

In the present study, we have demonstrated that HG-treated H9C2 cells are more vulnerable to H/R stimulation, which is associated with excessive oxidative stress. Treatment with glycine could protect H9C2 cells from HG- and H/R-induced injury, which was possibly associated with improvement of mitochondrial quality and inhibition of PKC*β*2 activation. To our knowledge, this is the first study to investigate the effectiveness of glycine supplementation in H9C2 cells exposed to HG and H/R stimulation.

Glycine, a fundamental element of glutathione that is the key endogenous antioxidant, is insufficiently synthesized under normal feeding conditions, particularly in a diseased state [14]. Several studies have suggested that glycine may be involved in regulating glucose homeostasis by stimulating glucagon and insulin release [18, 19]. Dietary glycine significantly blunted the characteristics of diabetes and delayed the progression of diabetic cataract and the development of hepatic steatosis; these protective effects of glycine are involved in the inhibition of oxidative stress [20, 21]. Thus, supplementation of glycine may have potential to prevented diabetic complications, including diabetic cardiomyopathy and ischemic heart disease. Increasing studies showed that glycine preconditioning attenuated cerebral and pulmonary IRI by inhibiting neuronal apoptosis and improving mitochondrial function, respectively [22, 23]. Besides, preadministration of glycine displayed protective effects on cardiomyocytes under H/R conditions and ex vivo hearts suffering from IRI [24]. In the present study, glycine treatment could attenuate HG- and H/R-induced H9C2 cellular damage, indicated by increased cell viability and decreased LDH release.

Normally, ROS can be eliminated efficiently by antioxidative enzyme system in mitochondrial matrix, but redundant ROS would exhaust endogenous antioxidant enzymes, such as glutathione, and this will induce oxidative stress injury and mitochondrial dysfunction, which in turn accumulates ROS [25, 26]. New research suggests that activation of these genes involved in mitochondrial glycine production conferred a restoration of age-associated mitochondrial respiration defects and a reduction of ROS accumulation; similar effects were shown in exogenous glycine supplement [16]. What is more, it has been certified that inhibition of glycine cleavage system is required for cancer cells to adapt to the hypoxia microenvironment [27]. That implied the key role of glycine in cellular redox homeostasis. Our result showed that glycine remarkably attenuated MDA production and increased SOD activity in H9C2 cells subjected to HG and H/R stimulation, as well as decreased MMP depolarization, which is implicated in mitophagy due to mitochondrial dysfunction [17]. Thus, glycine may diminish HG- and H/R-induced oxidative stress injury via improving mitochondrial function. This is in keeping with recent advance that mitochondrial-targeted antioxidant therapy could inhibit ROS production and increase cryopreserved sperm viability [26].

Mitophagy, a specific type of autophagy, is an essential process of mitochondria quality control eliminating damaged mitochondria and performed by members of a mitochondrial molecular ensemble, including mitofusins (Mfn), Cyt-C, and LC-3 [28]. Deficiency of mitophagy may result in the failure of mitochondria quality control under stressed conditions such as IRI and diabetes [29]. It is well known that PINK1/Parkin signaling is the classical mediator of mitophagy; however, emerging review suggested that the colocalization of PINK1 and Mfn-2 at the outer mitochondrial membrane provided an opportunity for PINK1 to phosphorylate Mfn-2, which is necessary for mitophagic Parkin binding to mitochondria [28]. In the present study, supplementary glycine suppressed the downregulation of Mfn-2 and LC-3II induced by HG and H/R, which might mean the reactivation of PINK1/Parkin-dependent mitophagy. Further, the repression of Cyt-C and restoration of MMP induced by glycine implied the improvement of mitochondrial quality.

In diabetic myocardium, excessive activation of PKC and redundant ROS induced by hyperglycemia have been implicated in the dysregulation of mitophagy [30], which has been linked closely with diabetic cardiac dysfunction and myocardial IRI [31, 32]. Pervious study indicated that PKC inhibition can relieve cigarette smoke extract-induced mitochondrial dysfunction in human airway smooth muscle cells via restoring Mfn-2 expression and decreasing ROS generation [33]. Downregulation of PKC*β*1/2 and upregulation of penumbra proteins involved in mitochondria quality control and mitophagy could be associated with tissue recovery and alleviation in penumbra around a photothrombotic infarction core in rat cerebral cortex [34]. Besides, selective inhibition of PKC*β*2 has been identified to ameliorate myocardial [35] and hepatic [36] IRI in diabetes via decreasing ROS production. Given the cytoprotection of mitophagy in IRI [37], it is reasonable that PKC*β*2 participated in mitophagic regulation during diabetic myocardial IRI. In the present study, similar to the treatment of PKC*β*2 inhibitor CGP, glycine treatment could attenuate PKC*β*2 activation, accompanied with increased Mfn-2 and LC-3II expression and decreased Cyt-C expression, which ultimately improved mitochondrial quality in H9C2 cells subjected to HG and H/R stimulation. Therefore, the beneficial effects of glycine are involved in the inhibition of PKC-*β*2 activation and improvement of mitochondrial quality.

In summary, the current results demonstrated that HG- and H/R-induced cell injury is associated with excessive oxidative stress and PKC-*β*2 activation. Supplementary glycine protects H9C2 cells from HG- and H/R-induced cellular injury by suppressing PKC-*β*2 activation and improving mitochondria quality. The current findings suggest that supplementary glycine and inhibition of PKC-*β*2 activation may be useful approaches for attenuating diabetes-related abnormalities and ischemic heart disease in diabetes. However, the potential mechanisms of glycine-induced cardioprotection against myocardial IRI in diabetes remain to be further addressed.
